# Mortality and Pulmonary Complications of Post-stroke Dysphagia: A Casuistic Review of an Acute Stroke Unit

**DOI:** 10.7759/cureus.74993

**Published:** 2024-12-02

**Authors:** Pedro M Coelho, Pedro L Almeida, Ilídia Carmezim, Andreia Silva, Rafaela Evangelista, Cláudia Dinis, Teresa Martins, Ana Torres, Ana Gomes, Jorge Caldas

**Affiliations:** 1 Physical Medicine and Rehabilitation, Unidade Local de Saúde (ULS) de Viseu Dão-Lafões, Viseu, PRT; 2 Stroke Unit, Unidade Local de Saúde (ULS) de Viseu Dão-Lafões, Viseu, PRT; 3 Physical Medicine and Rehabilitation, Unidade Local de Saúde (ULS) do Viseu Médio Tejo, Tomar, PRT; 4 Physical Medicine and Rehabilitation, Unidade Local de Saúde (ULS) de Coimbra, Coimbra, PRT

**Keywords:** dysphagia, mortality, rehabilitation, respiratory tract infection, stroke

## Abstract

Introduction: Dysphagia is a common post-stroke neurological disorder. Early screening for dysphagia can identify patients at risk of aspiration, thereby reducing the occurrence of pulmonary complications, morbidity, and mortality in this population.

Objectives: This study aims to evaluate the impact of an intervention in a stroke unit, following a retrospective study carried out in the same unit in 2020, which investigated the association between dysphagia and acute cerebrovascular disease and analyzed the prevalence of readmissions due to respiratory tract infections (RTI) and mortality. An assessment of the factors related to a higher risk of developing post-stroke dysphagia was also performed.

Material and methods: A retrospective observational study analyzed 210 clinical records of patients with acute cerebrovascular disease, including clinical history, neurological examination, imaging, and Gugging Swallowing Screen in the initial 48 hours. Patient follow-up for three months through medical records was used to evaluate RTI and mortality.

Results: Among the 210 clinical records examined, 209 (99.5%) underwent dysphagia assessment, contrasting with the previously reported casuistic from this unit (40.8%). The prevalence of dysphagia was also higher (50.7% vs. 32.4%). Over a three-month follow-up, RTI occurred in 19% of all patients, in 35.8% of all dysphagic patients, and in 67.7% (p<0.001) of those with severe dysphagia. The overall three-month mortality rate was 9.0% and 12.3% in dysphagic patients, particularly in patients with severe dysphagia (29.0%; p<0.001). Compared to the previous report, this study found a decrease of 1.4% in all-cause mortality (9.0% vs. 10.4%), 11.9% in all-cause mortality in dysphagic patients (12.3% vs. 24.2%), and 46% in all-cause mortality in patients with severe dysphagia (29.0% vs. 75.0%). A higher risk of dysphagia was significantly associated with older age (p<0.001), female gender (p<0.006), severe stroke (p<0.001), hemorrhagic stroke (p=0.005), strokes involving the carotid territory (p=0.040), dysarthria (p=0.004), aphasia (p<0.001), and type of aphasia, particularly global and Broca's aphasia (p=0.022).

Conclusions: The recent rates of all-cause mortality at the three-month follow-up, especially in the severe dysphagia group, indicate an overall improvement in the quality of patient care in the stroke unit intervened with regard to dysphagia, while the higher prevalence of RTI post-stroke at three months may not only reflect the larger number of patients screened for dysphagia but also aging, multimorbidity, and the increasing incidence of stroke on the Portuguese population. The type of cerebrovascular disease, vascular territory, age, gender, National Institutes of Health Stroke Scale and Glasgow Coma Scale scores, dysarthria, aphasia, and type of aphasia were significant associated factors to post-stroke dysphagia. The intervention of a multi-professional team with the implementation of a protocol for early dysphagia screening is crucial to optimizing the outcomes of patients with post-stroke dysphagia.

## Introduction

Dysphagia is among the potential neurological deficits resulting from a stroke, and according to the literature, it can affect up to 78% of stroke survivors [1]. This incidence may be influenced by the type and severity of stroke, history of stroke, and individual patient characteristics, including comorbidities like diabetes mellitus. On the other hand, the reported incidence of post-stroke dysphagia varies depending on the type of swallowing assessment test employed and the timing of its application after the event [1,2].

The neural circuit responsible for initiating the swallowing process is located in the rostral part of the medulla oblongata. It can be activated by stimuli originating from various cortical and subcortical areas. Consequently, strokes affecting any of the hemispheres, subcortical structures, or the brainstem can result in dysphagia [3].

Post-stroke dysphagia is associated with malnutrition, dehydration, and pulmonary complications, particularly pneumonia, which in turn leads to prolonged hospitalization, dependency, and increased mortality [4,5]. Pneumonia is a frequent post-stroke complication, with multifactorial pathophysiology and a reported incidence of 14% [5]. The impaired motor control of swallowing observed in patients with dysphagia facilitates the aspiration of food and/or liquid residues into the lower respiratory tract, contributing to the development of pneumonia. In these patients, the associated protective airway responses like coughing may be absent, which poses a challenge and delays the diagnosis [4]. A nasogastric tube (NGT) is often placed to allow feeding. However, the presence of an NGT, particularly when coupled with inadequate oral hygiene, can predispose individuals to alterations in oropharyngeal bacterial flora, biofilm formation, mechanical disruptions of clearance mechanisms, and increased gastro-oesophageal reflux due to impaired oesophageal sphincter function. In addition, there may be immunosuppression mediated by overactivation of the sympathetic nervous system and the hypothalamic-pituitary-adrenal axis, especially in strokes of greater severity [5]. An altered state of consciousness may also contribute to aspiration [2,5].

Patients with post-stroke dysphagia, particularly those without an early screening, face a three to four times higher risk of developing pneumonia [1]. This rises further to approximately 11 times in patients with confirmed aspiration [1,5].

Diagnostic tests such as videofluoroscopy (VF) and fiberoptic endoscopic evaluation of swallowing provide a detailed assessment of the entire swallowing process. However, these tools are not suitable for the acute phase of stroke due to their unavailability in that setting and the necessity of patient cooperation [1]. This reinforces the importance of establishing a dysphagia screening protocol and initiating interventions to minimize the risk of aspiration in patients with identified dysphagia. Recent international stroke guidelines advocate the early implementation of a dysphagia screening protocol, preferably conducted before any oral intake of food or liquids, by a trained healthcare professional [6,7]. To date, there is no consensus on the best screening test. A 2021 Cochrane review analyzed 37 screening tests for post-stroke dysphagia to assess their sensitivity and specificity. This review indicated that screening tests involving water in combination with other consistencies were more accurate than tests using only water or other methods. Among the former category, the Gugging Swallowing Screen (GUSS) exhibited the best overall performance. The authors further concluded that tests administered by nurses outperform those applied by other healthcare professionals, excluding speech-language pathologists (SLPs) [1,8].

Therapeutic options for the management of dysphagia include hygieno-dietetic, postural, behavioral, pharmacological, and neurostimulation interventions, although, at the moment, there is not enough data to establish specific recommendations [9,10].

In 2020, a retrospective study was carried out in the same stroke unit where the present study was conducted. The purpose of the previous research was to investigate the relationship between dysphagia and the type of stroke, cerebral hemisphere involved, severity on admission, concomitant language changes, and long-term complications (readmissions due to pulmonary complications and mortality). The results were presented to the stroke unit's healthcare professionals in order to raise awareness of this issue and implement a protocol for dysphagia screening. Therefore, the current study aimed to assess the clinical outcomes of patients admitted to this stroke unit after the aforementioned intervention in order to evaluate its impact. An assessment of the factors related to a higher risk of developing post-stroke dysphagia was also performed.

## Materials and methods

Subjects and procedure

The present observational retrospective study was carried out between October 2021 and March 2022, focusing on the clinical records of all patients admitted to the stroke unit with a diagnosis of acute cerebrovascular disease. Specific exclusion criteria were established: (1) patients under 18 years old were excluded, as they are not typically allocated to stroke unit care; (2) previous mRS score of ≥3; and/or (3) preexisting dysphagia. This approach aimed to ensure the selection of the most independent and cooperative patients, isolate the variable under study (dysphagia), and minimize the impact of any confounding variables.

In order to estimate all-cause mortality and readmissions related to pulmonary complications, all patients included in this study had follow-up medical records in the hospital’s software (consultations, admissions, and hospitalizations) during the three months following the cerebrovascular event.

Assessment of swallowing function was performed within 48 hours of admission using GUSS. This scale has a reported sensitivity of 100% (95% CI 77-100%) and specificity of 69% (95% CI 41-89%) and has been validated as a bedside dysphagia screening test for acute stroke patients [1]. GUSS involves an initial evaluation of indirect swallowing function through a simple swallowing test, followed by direct swallowing tests consisting of sequential subtests evaluating semisolid, liquid, and solid swallowing trials. Patients progress to the next subtest only upon achieving the maximum score in the previous one [11]. The severity of dysphagia was categorized into four groups: severe (0-9 points), moderate (10-14 points), mild (15-19 points), and no dysphagia (20 points). Mild and moderate dysphagia groups were combined for statistical analysis.

Demographic data encompassing age and gender were collected. The admission records of the patient’s clinical history, physical exam, including neurologic examination, evaluation of the Glasgow Coma Scale (GCS) and National Institutes of Health Stroke Scale (NIHSS) in the emergency room, and brain imaging (brain CT scans and CT angiography of the cerebral arteries) were used to establish the diagnosis. The types of acute cerebrovascular disease considered were ischemic stroke, hemorrhagic stroke, transient ischemic attack (TIA), and cerebral venous sinus thrombosis (CVST). Vascular territory (carotid or vertebrobasilar) and brain hemispheric location (right, left, or bilateral) of the lesion were accessed through imaging exams. The level of consciousness was based on the GCS score. Communication disorders (dysarthria or aphasia) were documented based on neurological examination records. The subtypes of aphasia considered were: global, Broca’s, Wernicke’s, conduction, anomic, transcortical motor, transcortical sensitive, and mixed transcortical. The diagnosis of respiratory tract infections (RTI) relied on clinical history, physical examination, analytical (elevated inflammatory markers), and chest radiography findings.

The study protocol received approval from the Institutional Review Committee of the Tondela-Viseu Hospital Center (approval number: 02/18/03/2022), aligning with the World Medical Association's Declaration. By assigning an alphanumeric code to each database record, patient privacy was safeguarded. The requirement for patient consent was dispensed by the review committee due to the nature of data collection. The reporting of this study adheres to the guidelines outlined in the STROBE statement.

Statistics

SPSS Statistics version 26.0 (IBM Corp. Released 2019; IBM SPSS Statistics for Windows, Version 26.0. Armonk, NY: IBM Corp.) was used to conduct statistical analysis. The normality of the data for each continuous variable was evaluated using the Kolmogorov-Smirnov test to determine whether parametric or nonparametric tests were appropriate. The continuous variables age, NIHSS, GCS, and GUSS did not follow a normal distribution (p<0.05). GUSS was transformed into an ordinal variable due to its skewed distribution. Thus, the Kruskal-Wallis nonparametric test was applied to compare dysphagia with age, NIHSS, and GCS. These data are presented as mean ranks.

Additionally, the associations between dysphagia and qualitative variables such as gender, type of cerebrovascular disease, vascular territory, involved hemisphere, dysarthria, aphasia and its subtypes, RTI at three months, and all-cause mortality at three months were explored through the Chi-square test. The information regarding these variables was presented using frequencies alongside their relative percentages. The level of significance was set at p<0.05.

## Results

Descriptive statistics

The current study encompassed 210 clinical records of patients diagnosed with acute cerebrovascular disease. Among these, one hundred and eight patients were male (51.4%), and 102 were female (48.6%). Ischemic stroke was the predominant cerebrovascular event, representing three-quarters (74.8%) of the sample, with a slightly higher prevalence in the left hemisphere compared to the right (56.7% and 43.3%, respectively). The carotid vascular territory was the most frequently involved (88.0%). CVST was observed in only one patient.

In this study, dysarthria and aphasia were present in more than half of the patients upon neurological examination. Within the aphasic subgroup, global and Broca's aphasia were the most prevalent types, accounting for 55.3% and 25.5%, respectively. The majority of patients had a GCS score of 15, while only 1.4% scored below 9. Out of 210 clinical records reviewed, it was observed that 209 patients (99.5%) underwent dysphagia assessment using the GUSS scale. Of those, 103 (49.3%) exhibited no signs of dysphagia, 75 (35.9%) had mild to moderate dysphagia, and 31 (14.8%) developed severe dysphagia. The overall prevalence of dysphagia in this study was 50.7%.

At three months of follow-up, the prevalence of RTIs and all-cause mortality were, respectively, 19% (n=40) and 9% (n=19). Both were higher in the severe dysphagia group. No patients were lost to follow-up during the time frame considered. The demographic and clinical characteristics of the included patients are outlined in Table 1.

**Table 1:** Description of qualitative variables TIA: transient ischemic attack, CVST: cerebral venous sinus thrombosis, VB: vertebrobasilar, RTI: respiratory tract infection, NIHSS: National Institutes of Health Stroke Scale, GCS: Glasgow Coma Scale, GUSS: Gugging Swallowing Screen

	Male (n = 108)	Female (n = 102)	Total (n = 210)
Age (years)			
≤45	3 (2.8)	4 (3.9)	7 (3.3)
46-60	17 (15.7)	3 (2.9)	20 (9.5)
61-75	36 (33.3)	27 (26.5)	63 (30.0)
>75	52 (48.1)	68 (66.7)	120 (57.1)
Type of cerebrovascular event			
Ischemic (%)	81 (75)	76 (74.5)	157 (74.8)
Hemorrhagic (%)	10 (9.3)	10 (9.8)	20 (9.5)
TIA (%)	17 (15.7)	15 (14.7)	32 (15.2)
CVST (%)	0 (0.0)	1 (1.0)	1 (0.5)
Hemisphere			
Right (%)	45 (42.1)	45 (44.6)	90 (43.3)
Left (%)	62 (57.9)	56 (55.4)	118 (56.7)
Territory			
Carotid (%)	92 (85.2)	92 (91.1)	184 (88.0)
VB (%)	16 (14.8)	9 (8.9)	25 (12.0)
Dysarthria			
Yes (%)	40 (37.0)	38 (37.3)	78 (37.1)
No (%)	68 (63.0)	64 (62.7)	132 (62.9)
Aphasia			
Yes (%)	19 (17.6)	28 (27.5)	47 (22.4)
No (%)	89 (82.4)	74 (72.5)	163 (77.6)
Type of aphasia			
Global (%)	10 (52.6)	16 (57.1)	26 (55.3)
Broca (%)	5 (26.3)	7 (25.0)	12 (25.5)
Wernicke (%)	2 (10.5)	3 (10.7)	5 (10.6)
Conduction (%)	0 (0.0)	1 (3.6)	1 (2.1)
Anomic (%)	1 (5.3)	0 (0.0)	1 (2.1)
Transcortical motor (%)	1 (5.3)	0 (0.0)	1 (2.1)
Transcortical sensitive (%)	0 (0.0)	0 (0.0)	0 (0.0)
Mixed transcortical (%)	0 (0.0)	1 (3.6)	1 (2.1)
NIHSS			
<5 (%)	74 (69.2)	46 (45.1)	120 (57.4)
5-22 (%)	31 (29.0)	52 (51.0)	83 (39.7)
>22 (%)	2 (1.9)	4 (3.9)	6 (2.9)
GCS			
<9	0 (0.0)	3 (2.9)	3 (1.4)
9-14	24 (22.2)	33 (32.4)	57 (27.1)
15	84 (77.8)	66 (64.7)	150 (71.4)
GUSS			
0-9 (%)	14 (13.1)	17 (16.7)	31 (14.8)
10-19 (%)	29 (27.1)	46 (45.1)	75 (35.9)
20 (%)	64 (59.8)	39 (38.2)	103 (49.3)
RTI at 3-month follow-up			
Yes (%)	19 (17.6)	21 (20.6)	40 (19.0)
No (%)	89 (82.4)	81 (79.4)	170 (81.0)
Death at 3-month follow-up			
Yes (%)	10 (9.3)	9 (8.8)	19 (9.0)
No (%)	98 (90.7)	93 (91.2)	191 (91.0)

Statistical analysis

The following associations were derived from the statistical analysis performed on data from the 209 patients who underwent dysphagia assessment with complete clinical records of GUSS evaluation. The findings are detailed in Tables 2-3.

**Table 2:** Summary of associations between dysphagia and quantitative variables Significant p-values are reported in bold. NIHSS: National Institutes of Health Stroke Scale, GCS: Glasgow Coma Scale

	Dysphagia (n = 106)		No dysphagia (n = 103)	p-value
	Severe (n = 31)	Mild to moderate (n = 75)		
Age (mean rank)	132.8	123.4	83.3	<0.001
NIHSS score (mean rank)	170.3	121.3	72.2	<0.001
GCS (mean rank)	56.8	97.1	125.3	<0.001

**Table 3:** Summary of associations between dysphagia and qualitative variables Significant p-values are reported in bold. TIA: transient ischemic attack, CVST: cerebral venous sinus thrombosis, VB: vertebrobasilar, RTI: respiratory tract infection

	Dysphagia (n = 106)			No dysphagia (n = 103)	p-value
	Severe (n = 31)	Mild to moderate (n = 75)			
Gender					0.006
Male (%)	14 (45.2)	29 (38.7)		64 (62.1)	
Female (%)	17 (54.8)	46 (61.3)		39 (37.9)	
Type of cerebrovascular event					0.005
Ischemic (%)	26 (83.9)	62 (82.7)		68 (66.0)	
Hemorrhagic (%)	5 (16.1)	7 (9.3)		8 (7.8)	
TIA (%)	0 (0.0)	6 (8.0)		26 (25.2)	
CVST (%)	0 (0.0)	0 (0.0)		1 (1.0)	
Territory					
Carotid (%)	31 (100.0)	67 (89.3)		85 (83.3)	0.040
VB (%)	0 (0.0)	8 (10.7)		17 (16.7)	
Hemisphere					0.159
Right (%)	17 (54.8)	35 (47.3)		38 (37.3)	
Left (%)	14 (45.2)	39 (52.7)		64 (62.7)	
Dysarthria					0.004
Yes (%)	14 (45.2)	37 (49.3)		27 (26.2)	
No (%)	17 (54.8)	38 (50.7)		76 (73.8)	
Aphasia					<0.001
Yes (%)	17 (54.8)	16 (21.3)		13 (12.6)	
No (%)	14 (45.2)	59 (78.7)		90 (87.4)	
Type of aphasia					0.022
Global (%)	15 (88.2)	7 (43.8)		3 (23.1)	
Broca (%)	1 (5.9)	4 (25.0)		7 (53.8)	
Wernicke (%)	0 (0.0)	3 (18.8)		2 (15.4)	
Conduction (%)	1 (5.9)	0 (0.0)		0 (0.0)	
Anomic (%)	0 (0.0)	1 (6.3)		0 (0.0)	
Transcortical motor (%)	0 (0.0)	0 (0.0)		1 (7.7)	
Transcortical sensitive (%)	0 (0.0)	0 (0.0)		0 (0.0)	
Mixed transcortical (%)	0 (0.0)	1 (6.3)		0 (0.0)	
RTI at 3-month follow-up					<0.001
Yes (%)	21 (67.7)	17 (22.7)		2 (1.9)	
No (%)	10 (32.3)	58 (77.3)		101 (98.1)	
Death at 3-month follow-up					<0.001
Yes (%)	9 (29.0)	4 (5.3)		6 (5.8)	
No (%)	22 (71.0)	71 (94.7)		97 (94.2)	

After acute cerebrovascular disease, the likelihood of dysphagia was higher in older patients (p<0.001). Additionally, female patients were more likely to experience dysphagia, particularly mild to moderate dysphagia (p<0.006).

The presence of dysphagia was also significantly related to the type of stroke (p=0.005). Among patients with acute ischemic stroke, nearly 56% exhibited dysphagia, with 39.7% displaying mild to moderate dysphagia and 16.7% exhibiting severe dysphagia. Similarly, in the hemorrhagic stroke group, 60% had dysphagia, 35% of those with mild to moderate dysphagia, and 25% with severe dysphagia. The majority of patients admitted with TIA did not develop dysphagia, even though 18.8% had mild to moderate dysphagia. The patient with CVST did not develop dysphagia.

Although a significant association between hemispheric location (right/left) and dysphagia was not found in this study, the affected vascular territory was shown to be related to the presence of dysphagia (p=0.040). In the majority of patients who developed dysphagia, the acute cerebrovascular event involved the carotid territory (92.5%). This observation was particularly evident in individuals with severe dysphagia, as all of them (100%) occurred in the carotid territory.

Among patients without dysphagia, 73.8% also had no dysarthria on admission. Of those with dysphagia, 48.1% also presented concurrent dysarthria. Patients with mild to moderate dysphagia manifested dysarthria on admission more often than those with severe dysphagia (p=0.004).

Furthermore, patients with aphasia on admission were more likely to exhibit severe dysphagia (p<0.001). Among patients who presented with both aphasia and dysphagia, global aphasia was the predominant type (p<0.022). Around 88.2% of patients with global aphasia also had severe dysphagia.

A significant relationship between dysphagia and NIHSS score was found (p<0.001). Patients with no dysphagia demonstrated the lowest scores in the NIHSS, while those with severe dysphagia had the highest scores. A similar association was noted between dysphagia and GCS on admission, with higher scores reported in the non-dysphagic group and lower scores in patients with severe dysphagia (p<0.001).

At three-month follow-up, the risk of RTI was significantly higher in patients with dysphagia, especially in the severe dysphagia group (p<0.001). All-cause mortality at three months was 12.3% in patients with dysphagia and 5.8% in patients without dysphagia. It was also superior in the severe dysphagia group compared with the mild to moderate dysphagia group (p<0.001).

## Discussion

Following an acute cerebrovascular event, dysphagia is common and presents in varying degrees of severity. The 50.7% prevalence of dysphagia in the present study was within the range reported in the literature, as it can affect up to 78% of patients who have had a stroke [1,2].

This study's results revealed significant associations between the presence of dysphagia and the demographic variables age (p<0.001) and gender (p<0.006). As anticipated, older individuals exhibited a higher risk of developing post-stroke dysphagia [2,12]. Although the current evidence regarding gender differences in the occurrence of post-stroke dysphagia remains heterogeneous [13,14], in this study, its prevalence was higher in women. A possible explanation for this result could be the later age at which women experience their first stroke, as older age constitutes a known risk factor for the development of post-stroke dysphagia [12,15,16].

Previous studies have also reported a higher incidence of dysphagia in intracerebral hemorrhage [12,17]. In the present study, the risk of dysphagia was significantly higher in hemorrhagic and ischemic strokes and slightly higher in hemorrhagic stroke (60% vs. 56%, p=0.005). The authors believe these prevalences are associated with greater severity of strokes, particularly the hemorrhagic type. This similar prevalence of dysphagia in both types of stroke, with a much less marked difference than reported in the literature [17] might be explained by the small sample size.

As previously described, ischemic strokes with involvement of the anterior circulation, such as the middle cerebral artery, are frequently related to the development of post-stroke oral phase dysphagia [18]. In this study, vascular territory was significantly associated with dysphagia (p=0.040). The carotid territory was most frequently involved among patients with dysphagia, especially in the severe dysphagia group. Similarly to previous research, no significant association between dysphagia and lesion hemispheric location (right/left) was found (p=0.159) [17].

Studies are highlighting the association between dysphagia and dysarthria or aphasia [19]. This finding was also observed, suggesting the influence of the stroke severity. A greater severity increases the likelihood of aphasia, and a lower severity increases the likelihood of dysarthria (p<0.001, p=0.004). The most common type of aphasia was global aphasia, followed by motor aphasia (Broca's aphasia), which is in accordance with the literature [20,21].

The NIHSS, a stroke severity assessment tool, has already been described as a predictor of dysphagia [2,12]. In spite of its limited sensitivity, which means that it should not be used by itself for initial management [22], a significant relationship was noted (p<0.001) between the NIHSS score and both the development and severity of dysphagia.

Aspiration, a major complication of dysphagia, is a contributing factor to stroke-associated pneumonia. This study revealed that the prevalence of RTIs at three-month follow-up was approximately nineteen times higher in dysphagic patients compared to the non-dysphagic group (35.8% vs. 1.9%, p<0.001), especially in patients with severe dysphagia.

A three-month post-stroke all-cause mortality of 12.3% in dysphagic patients was also found, which is similar to that reported in the literature [23], with the highest contribution from the severe dysphagia group (29.0%, p<0.001).

Since the study conducted at this same stroke unit between October 2019 and March 2020 by Silva et al. [24], the GUSS scale, performed by trained nursing staff, remains the only routine method for dysphagia screening in post-stroke patients. A diagnostic tool for the assessment of dysphagia, like VF, is still not available in this unit, which in addition to the retrospective design of this study, continues to be an important limitation.

Silva et al. [24] reinforced the need to review the dysphagia approach protocol for patients admitted to the stroke unit. Previously, the lack of nursing staff with specific training in dysphagia screening resulted in inefficient dysphagia screening and management with a small fraction of patients being evaluated. The protocol, established in 2016 by a collaborative effort involving a multi-professional rehabilitation team, has progressively expanded to all beds in the stroke unit. This expansion considered the gradual proficiency development of the nursing staff and the necessity for supervision by specialized members. Furthermore, an annual theoretical training course on dysphagia assessment is conducted to address challenges and dispel any lingering uncertainties. These yearly sessions not only facilitate the integration of new nursing staff into the stroke unit but also equip them with the skills to implement the protocol. As part of the reorganization, in addition to the initial assessment, the plan of interventions for these patients has been revisited and adjusted according to the severity of dysphagia. Nil per os diet until completion of the dysphagia screening, patient positioning (sitting/fowler), physical examination of the patient's oropharyngeal structures, continuous monitoring of pulse oximetry during dysphagia assessment using the GUSS scale, and reassessment by different professionals in dubious cases are among the measures incorporated in the updated protocol. At discharge, the nursing staff, SLPs, and physiatrists provided training and detailed information to patients and family or caregivers about how to feed patients with dysphagia. The physiatrist also referred patients to speech and language therapy in ambulatory care or to external rehabilitation institutions when applicable. It's crucial to highlight that the revision of this protocol was a collaborative effort involving a multidisciplinary team, which included physicists, internists, nurses, and SLPs. Physiatrists are responsible for the functional assessment of all stroke patients and the coordination of the articulation between all rehabilitation team members, comprising rehabilitation nurses, SLPs, physiotherapists, and occupational therapists.

A comparison of relevant data between the present study and the one by Silva et al. is outlined in Table 4.

**Table 4:** Comparison of results from Silva et al. [24] and Coelho et al. Relevant values are highlighted in bold. RTI: respiratory tract infection, GUSS: Gugging Swallowing Screen

	Silva et al. [24] (Oct/2019-Mar/2020)	Coelho et al. (Oct/2021-Mar/2022)
Dysphagia screening (%)	40.8	99.5
Overall dysphagia (%)	32.4	50.7
GUSS		
0-9 (%)	7.8	14.8
10-19 (%)	24.5	35.9
20 (%)	67.7	49.3
RTI at 3-month follow-up		
Overall (%)	2.4	19.0
No dysphagia	-	1.9
Dysphagia (%)	-	35.8
Severe dysphagia (%)	-	67.7
Death at 3-month follow-up		
Overall (%)	10.4	9.0
No dysphagia	1.4	5.8
Dysphagia (%)	24.2	12.3
Severe dysphagia (%)	75.0	29.0

When contrasting the results of both studies, conducted on a comparable sample within a similar timeframe, an overall decrease of 1.4% (9.0% vs. 10.4%) in all-cause mortality of patients with acute cerebrovascular disease is observed at three-month follow-up [24]. Furthermore, there is an 11.9% decrease (12.3% vs. 24.2%) in all-cause mortality among patients identified with dysphagia at the three-month follow-up, particularly noticeable within the severe dysphagia group, demonstrating a 46% reduction (29.0% vs. 75.0%). Another relevant point is that nearly all (99.5%) of the patients admitted to the stroke unit underwent dysphagia evaluation using GUSS, as opposed to the previously mentioned study where only 40.8% were assessed. These numbers may reflect not only an effort to enhance the consistency and systematization of dysphagia screening in these patients but also an improvement in the overall quality of patient medical and nursing care. The overall prevalence of dysphagia was higher (50.7% vs. 32.4%), probably due to the higher number of patients screened. The readmission rate due to RTIs at three-month follow-up among patients evaluated for dysphagia was 18.1%, whereas Silva et al. reported 2.4% [24]. This may be interpreted considering that almost all patients were screened for dysphagia and the number of stroke survivors with severe dysphagia was 7% superior when compared with the aforementioned study (14.8% vs. 7.8%) [24]. Furthermore, the majority (67.7%) of the patients readmitted due to RTIs at the three-month follow-up belonged to the severe dysphagia group.

The post-stroke RTI prevalence at three-month follow-up in the present study is higher than current literature reports (9.4%) [25]. The authors took several aspects into account. On one hand, cerebrovascular disease, mainly stroke, is the leading cause of mortality, morbidity, and disability in Portugal [26]. On the other hand, across the EU member states, Portugal has one of the highest percentages of elderly people and also one of the highest prevalences of multimorbidity among European countries [27,28]. A high prevalence of stroke in an aged and multimorbid population may explain the higher three-month follow-up post-stroke RTI prevalence in this study.

Dysphagia screening, prior to oral intake, for all stroke patients, regardless of stroke severity, is recommended by the American Heart Association/American Stroke Association [6,7]. Implementing oral hygiene protocols is also highly recommended to reduce the risk of stroke-associated pneumonia [7].

While SLPs are typically recognized as the most suitable professionals for the initial assessment of dysphagia, their availability in the acute context is limited. The nursing team seems to be the best alternative, according to a 2021 Cochrane review that concluded that dysphagia screening tools carried out by nurses performed consistently better compared to tests carried out by other healthcare professionals (excluding SLPs) [1]. Therefore, it is essential that these staff members continue to receive specialized training to ensure effective dysphagia screening and management [6].

As previously noted, NGT is often placed to feed patients with post-stroke dysphagia, although there are some complications associated with this feeding modality, namely malnutrition and RTIs [5]. Recent literature suggests that intermittent oro-esophageal tube feeding (IOE) may be a feasible alternative to NGT, with some of the advantages being the short duration of tube placement, the reduced risk of gastroesophageal reflux, bacterial colonization, skin or mucosal injury, and improvement of swallowing function through stimulation by the tube insertion [29,30]. IOE has demonstrated significantly better results regarding nutritional status, swallowing function, stroke-associated pneumonia, and depression compared to NGT, although multicentric studies with larger samples are needed [30].

## Conclusions

The reduction in three-month all-cause mortality, particularly in patients with severe post-stroke dysphagia, suggests an improvement in dysphagia screening performance and also in the overall quality of patient medical and nursing care in the stroke unit intervened, while the higher prevalence of RTIs at three-month follow-up in comparison to reported casuistics from this unit may not only reflect a significant increase in the number of patients screened for dysphagia but also aging, multimorbidity, and the increasing incidence of stroke on the Portuguese population. In this study, dysphagia was significantly associated with factors such as the type of cerebrovascular disease, vascular territory, age, gender, NIHSS and GCS scores, dysarthria, aphasia, and type of aphasia.
